# Optimising orbit counting of arbitrary order by equation selection

**DOI:** 10.1186/s12859-018-2483-9

**Published:** 2019-01-15

**Authors:** Ine Melckenbeeck, Pieter Audenaert, Thomas Van Parys, Yves Van De Peer, Didier Colle, Mario Pickavet

**Affiliations:** 1 Ghent University - imec, IDLab, Technologiepark 15, Ghent, 9052 Belgium; 2 Bioinformatics Institute Ghent, Ghent University, Ghent, Belgium; 3 Department of Plant Systems Biology, VIB, Technologiepark 927, Ghent, 9052 Belgium; 4 Department of Plant Biotechnology and Bioinformatics, Ghent University, Technologiepark 927, Ghent, 9052 Belgium; 5 Department of Biochemistry, Genetics and Microbiology, University of Pretoria, Pretoria 0028, South Africa

**Keywords:** Graph theory, Graphlets, Orbits, Equations, Optimisation, Cytoscape app

## Abstract

**Background:**

Graphlets are useful for bioinformatics network analysis. Based on the structure of Hočevar and Demšar’s ORCA algorithm, we have created an orbit counting algorithm, named Jesse. This algorithm, like ORCA, uses equations to count the orbits, but unlike ORCA it can count graphlets of any order. To do so, it generates the required internal structures and equations automatically. Many more redundant equations are generated, however, and Jesse’s running time is highly dependent on which of these equations are used. Therefore, this paper aims to investigate which equations are most efficient, and which factors have an effect on this efficiency.

**Results:**

With appropriate equation selection, Jesse’s running time may be reduced by a factor of up to 2 in the best case, compared to using randomly selected equations. Which equations are most efficient depends on the density of the graph, but barely on the graph type. At low graph density, equations with terms in their right-hand side with few arguments are more efficient, whereas at high density, equations with terms with many arguments in the right-hand side are most efficient. At a density between 0.6 and 0.7, both types of equations are about equally efficient.

**Conclusions:**

Our Jesse algorithm became up to a factor 2 more efficient, by automatically selecting the best equations based on graph density. It was adapted into a Cytoscape App that is freely available from the Cytoscape App Store to ease application by bioinformaticians.

## Background

The small-scale structure of a graph contains important information about that graph’s function. *Network motifs* [1] are defined as subgraphs of a larger graph that appear significantly more often in that large graph than would be expected in a purely random graph with the same number of nodes and edges. It was found that motifs can easily be used to distinguish networks of different functions from each other, like genetic transcription networks from ecosystem food webs [1]. To know whether a certain subgraph is a motif in a specific explored graph, its expected number of appearances in random graphs should be known. This means the subgraph must be counted in a large number of random graphs [1]. This may be sped up by sampling [2] or parallellisation [3], but the random graphs are still needed.

**Fig. 1:**
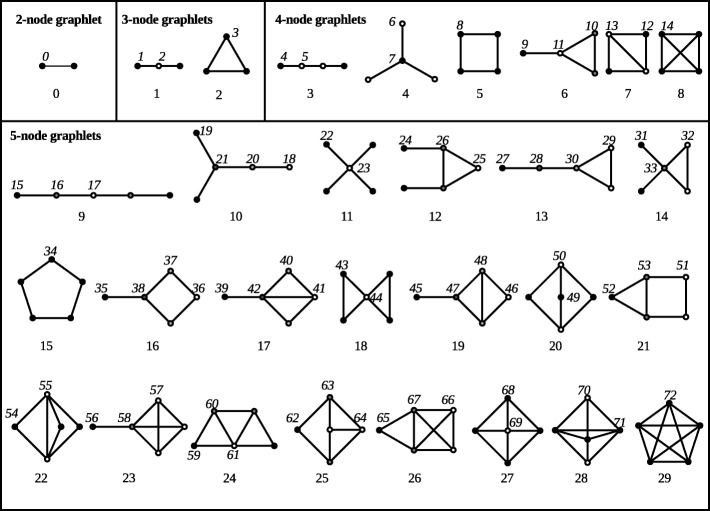
All graphlets of 2-5 nodes with their original numbering. Within each graphlet, grayscales show the graphlets’ orbits. Orbit numbers are shown close to a node of that orbit. Figure adapted from [10]

It was first proposed in [4] not to restrict interest to those subgraphs that appear more often than expected. Instead, PPI networks were analysed by counting all small connected, induced subgraphs. These subgraphs are called *graphlets*. All graphlets on 2 to 5 nodes are shown in Fig. 1. They are all assigned a unique identification number, which can be used to refer to that specific graphlet (shown under each graphlet in Fig. 1). Originally, graphlets were restricted to 5 or fewer nodes because of limitations in computing power. Later research [5, 6] increased that number of nodes. Regardless of the exact computing power available, the exponentially exploding number of graphlets enforces the use of some cut-off on the number of nodes in a graphlet. Therefore, if the term “all graphlets” is used in this paper, it means “all graphlets on *k* or fewer nodes”, with *k* some arbitrary but fixed cut-off.

Each graphlet’s nodes can be subdivided into symmetrically equivalent sets, called *orbits* [7]. For example, consider graphlet 1, the three-node path, in Fig. 1. By mirroring the graphlet, the two outside nodes can be swapped, which does not change the graphlet’s structure. Therefore, these two nodes are contained in the same orbit. The middle node can never be interchanged with either of the outside nodes without changing the graphlet’s structure. This means that this node is in a different orbit. In Fig. 1, the outer nodes of graphlet 1 are coloured black, whereas the middle node is coloured white, to indicate their respective orbits. Likewise, in all other graphlets in Fig. 1, the nodes are shaded according to their orbit. Like graphlets, orbits are numbered. These numbers are unique across all graphlets, so an orbit number can be used to identify both the orbit and its graphlet. In Fig. 1, the number of each orbit is indicated close to one of its nodes.

When a graphlet is found in an explored graph, it is possible to determine which graph node is in what orbit of that graphlet. That orbit is then said to *touch* that node. The number of times orbit *i* touches node *v* is called *v*’s *i*th *graphlet degree* [7]. Graphlet degrees are an extension of a node’s degree, which itself is equivalent to the 0th graphlet degree, i.e. the graphlet degree of the 2-node graphlet. As an extension of a graph’s degree distribution, the distribution of all of the graphlet degrees can be calculated, which is called the graph’s *graphlet degree distribution* or *GDD*. These graphlet degree distributions themselves are a useful tool for network comparison [7] and identification of nodes’ function [8].

To calculate a graph’s GDD, it seems at first sight that all graphlets within that graph need to be found. However, it was proved in [9] that this is not the case. They showed that, to calculate a node’s graphlet degrees (using graphlets on up to *k* nodes), a system of linear equations can be composed from all graphlets on *k*−1 nodes and all common neighbours of up to *k*−2 nodes in the explored graph. Solving this system of equations then results in the node’s graphlet degrees. Using equations allows part of the calculation to be done in advance and reused while counting multiple different orbits.

The ORCA algorithm (ORbit Counting Algorithm) [9] is the fastest available algorithm to calculate all nodes’ graphlet degrees. ORCA can count the orbits of graphlets up to either 4 or 5 nodes and uses such a system of equations to reduce this to finding graphlets on 3 or 4 nodes, respectively.

The *Jesse* algorithm, which managed to automatically generate equations for orbits of arbitrary order, as well as use them to count those orbits, was described in [5]. To this end, a non-ambiguous, extendable graphlet and orbit numbering was introduced. This numbering does not follow the original numbering in Fig. 1, but is constructed in an algorithmical, consistent way. Likewise, a novel type of graphlet that represents a specific orbit was introduced. In these graphlets, a single node is marked, which singles out that node’s orbit. These so-called *orbit representatives* were used as the nodes of a directed tree, in which the arcs were the addition of nodes and edges to these orbit representatives. This tree can then be used to find orbit representatives on *k*−1 nodes within a graph, after which automatically generated [10] equations are used to calculate the number of orbits of graphlets on *k* nodes.

However, many redundant equations are generated, so a large linearly dependent system of equations arises. Originally, the equations used in Jesse were chosen on a first-come-first-served basis. If Jesse generated more than 1 equation that could be used to count orbit *i*, only the one that was generated first was saved and used. All other generated equations are just as correct, though.

Once the system of equations is completed, it can be straightforwardly solved as its left-hand side, where graphlet degrees are related to each other, is an upper triangular matrix. However, filling in the right-hand side of all equations is more time-consuming, as this depends on the graph’s structure. Therefore, different, but equally correct sets of equations can be used and have an impact on Jesse’s running time.

Other techniques to count graphlets have been developed: ranging from combinatorial counting that uses graphlets’ symmetries and substructures to count them without finding any graphlet, which is currently limited to graphlets on 4 nodes [11], to sampling techniques that do not count all graphlets but identify randomly sampled subgraphs [6], or incremental counting [12].

In this paper, we investigate whether it is possible to select a set of equations that allows the graphlet degrees to be calculated faster than when using other sets, and whether this set depends on the explored graph’s properties. First, a number of graph theory terms need to be defined. A high-level explanation of Jesse’s internal structure follows. Then, the structure of a graphlet counting equation is explained, and the variable properties of equations are identified. With all of these, the effect of equation selection on Jesse’s speed can be investigated, accounting for different graph types, orders and sizes. Finally, the Cytoscape version of Jesse will be presented.

### Formal graph theory definitions

This section is a collection of formal definitions of terms which will be used later on in the paper. These are formal definitions for all terms used in this paper, but for more information on how and why these terms were constructed we refer to [10].

#### **Definition 1**

An *undirected graph*

$$G = (V,E),$$

where *V* is the collection of *nodes* and *E* is the collection of *edges* of *G*, such that

$$E \subseteq \{\{v,w\} | (v, w \in V \wedge v \neq w)\}.$$

Similarly, *V*(*G*) and *E*(*G*) are used to indicate *G*’s node and edge set, respectively. *n*=|*V*| is called the graph’s order, while *m*=|*E*| is called its size.

#### **Definition 2**

A graph *G*’s *density*

$$D(G) = \frac{|E|}{\binom{|V|}{2}} = \frac{2|E|}{|V|(|V|-1)}.$$

As it is the graph’s size divided by the maximal size that graphs of that order can have,

$$0 \leq D(G) \leq 1.$$

#### **Definition 3**

The *isomorphisms* between two graphs *G* and *H* are the one-to-one functions that map *G*’s nodes to *H*’s nodes, such that their edge sets are mapped onto each other as well.

$$\begin{aligned}{}\text{Iso}(G,H) &= \{f:{V}(G) \rightarrow {V}(H)|(f \text{ is bijective }\\ &\quad \wedge (\forall v, w \in {V}(G): (\{v,w\}\\&\quad \in {E}(G) \iff \{ f(v), f(w) \} \in {E}(V))))\}. \end{aligned} $$

Two graphs are said to be *isomorphic* if they have at least one isomorphism.

$$G \simeq H \iff \text{Iso}(G,H) \neq \emptyset $$

#### **Definition 4**

The *automorphisms* of a graph *G* are the isomorphisms of *G* to itself.

$$\text{Aut} (G) = \text{Iso}(G,G)$$

#### **Definition 5**

A graph’s nodes have a unique, ordered index:

$$\text{Ind} (v) = \text{Ind} (w) \iff v = w. $$

This index is chosen arbitrarily at graph creation. As a form of shorthand, we note *v*<*w* for Ind(*v*)<Ind(*w*).

#### **Definition 6**

The *induced subgraph* on a subset *V*_*s*_⊆*V*(*G*)of a graph *G*’s nodes is the graph formed by *V*_*s*_ and all edges between those nodes that are present in *G*.

$$G[V_{s}] = (V_{s}, \{ \{v,w\} \in E(G) : v, w \in V_{s}\}) $$

#### **Definition 7**

The *number of common neighbours* of a subset *V*_*s*_⊆*V*(*G*) of a graph *G*’s nodes will be noted as *c*(*V*_*s*_).

$$c(V_{s}) = | \{v \in V(G) |(\forall w \in V_{s} : \{v,w\} \in E(G))\} | $$

Following [9], *c*({*u*,*v*,...}) will also be noted as *c*(*u*,*v*,...).

#### **Definition 8**

A *graphletG* is a connected graph. Graphlets are assigned an unique numbering, such that the notation *G*_*i*_ denotes a unique graphlet for each value of *i*∈*ℕ*. A *k-graphlet* is a graphlet with *k* nodes.

#### **Definition 9**

The *instances* of a graphlet *G*_*i*_ in a graph *H* are the induced subgraphs of *H* that are isomorphic with *G*_*i*_.

#### **Definition 10**

The *automorphism orbit*, or *orbit* in short, of a node *v* in a graphlet *G*_*i*_ is the set of nodes to which *v* can be mapped by an automorphism of *G*_*i*_.

$$\text{Orb}(G_{i},v) = \left\{w \in V(G_{i}) | \left(\exists f \in \text{Aut} (G_{i}) : f(v) = w \right)\right\} $$

Orbits are assigned a unique numbering, spanning over all graphlets, such that the notation Orb_*j*_ can be used to identify both the orbit and its graphlet.

#### **Definition 11**

An *orbit representative* is a graphlet with a marked node *x*.

$$\Omega = (V,E,x) $$

with

$$x \in V \wedge E \subseteq \{\{v,w\} | (v, w \in V \wedge v \neq w) \} $$

Orbit representatives follow the same numbering as orbits, such that *Ω*_*i*_ is the orbit representative that corresponds with Orb_*i*_. The marked node of an orbit representative *Ω* will be noted as *x*(*Ω*).

#### **Definition 12**

The isomorphisms between two orbit representatives *Ω* and *Ψ* are the isomorphisms of *Ω* and *Ψ*’s graphlets that map *Ω*’s marked node to *Ψ*’s marked node.

$$\begin{aligned} \text{Iso}(\Omega,\Psi) &= \{f:{V}(\Omega) \rightarrow {V}(\Psi) |(f \text{ is bijective} \\ &\quad \wedge (\forall v, w \in {V}(\Omega): (\{v,w\}\\ &\quad \in {E}(\Omega) \iff \{ f(v), f(w)\} \in E(\Psi)))\\ &\quad \wedge (f({x}(\Omega)) = {x}(\Psi)))\} \end{aligned} $$

Two orbit representatives are said to be isomorphic if they have at least one isomorphism.

$$\Omega \simeq \Psi \iff \text{Iso}(\Omega,\Psi) \neq \emptyset $$

#### **Definition 13**

The automorphisms of an orbit representative *Ω* are the isomorphisms of *Ω* to itself.

$$\text{Aut}(\Omega) = \text{Iso}(\Omega,\Omega) $$

#### **Definition 14**

The *induced orbit representative* on a subset *V*_*s*_⊆*V*(*G*) of a graph *G*’s nodes and a marked node *x*∈*V*_*s*_ is the orbit representative formed by *V*_*s*_ and all edges between those nodes that are present in *G*, with *x* as the orbit representative’s marked node.

$$G[V_{s}, x] = \left(V_{s}, \left\{ \{v,w \} \in E(G) : v, w \in V_{s} \right\}, x \right) $$

#### **Definition 15**

The *instances* of an orbit representative *Ω*_*i*_ in a graph *H* are the induced subgraphs of *H* that are isomorphic with *Ω*_*i*_.

An orbit Orb_*i*_*touches* a node *v* in a graph *G* if its corresponding orbit representative *Ω*_*i*_ has an instance in *G* in which *v* is the marked node.

#### **Definition 16**

The *ith graphlet degree* *o*_*i*_(*v*)of a node *v* in a graph *G* is the number of times orbit *i* touches *v*.

$$o_{i}(v) = | \{S \subseteq V(G) : G[S,v] \simeq \Omega_{j} \} | $$

Once more, we refer the interested reader to [10] for more information.

## Implementation

In this section, the structure of our Jesse program will be explained in broad strokes. Afterwards, the concept of equation selection, as well as the factors that can be used to select equations, are explained. Finally, the different random graph types that were used in testing are described.

### Jesse

Jesse is the program we created to solve the orbit counting problem. It is described in detail in [5], but a short summary of the technique follows here.

The structure of Jesse’s main search algorithm is similar to ORCA’s structure: it is a tree-based graphlet search, followed by filling in and solving a system of equations relating the graphlet degrees to common neighbours of the found graphlets. There is one important difference with ORCA: every required structure can be generated for graphlets of theoretically unlimited order.

The most important datastructure is the orbit tree. This tree’s nodes are the orbit representatives and its directed arcs symbolize the addition of a node or edge to an orbit representative. With this orbit tree, finding instances of orbit representatives within a graph becomes a tree-walking matching algorithm.

Likewise, the orbit counting equations are generated. Because these equations relate the graphlet degrees to the number of common neighbours of orbit representative instances, those common neighbours need to be counted. This is done before the actual graphlet search, to allow for maximal re-use of calculations.

Then, the orbit representatives on (*k*−1) nodes are searched with the tree; with each found instance, the corresponding equations are filled in with the appropriate common neighbours. After the orbit search, the system of equations is solved. Interested readers are refered to [5] for more information.

### Equation selection

For each graphlet order, many more equations are generated than there are orbits that need to be counted. All of these equations are correct. They form a huge, linearly dependent, upper triangular system, which can be easily simplified to a maximal linearly independent system by discarding all equations but one for each orbit.

As an illustration, here are the 3 possible equations to count orbit 40.

1 $$ o_{40}(x) + 4o_{54}(x) = \sum\limits_{u,v,t: P_{6} (x,u,v,t)} [\!c(x, u, v) + c(x, u, t)]   $$

2 $$ o_{40}(x) + 2o_{57}(x) + o_{59}(x) + 2o_{66}(x)\! =\! \sum\limits_{u,v,t: P_{10} (x,u,v,t)} [\! c(u, t)\! - 1)]   $$

3 $$ \begin{aligned} o_{40}(x) &+ 4o_{54}(x) + o_{59}(x) + o_{60}(x) + 4o_{65}(x) + 2o_{66}(x)\\ & + 2o_{68}(x) + 6o_{70}(x) = \\ &\sum\limits_{u,v,t: P_{12} (x,u,v,t)} \left[(c(u) - 3) + (c(t) - 3) \right]  \end{aligned}  $$

in which

$$P_{6} (x,u,v,t) \equiv \!(G[\{x,u,v,t\},x] \simeq \Omega_{6} \wedge \{x,u\}\! \in E(G) \wedge v<\! t) $$

$$\begin{aligned} P_{10} (x,u,v,t) &\equiv (G[\{x,u,v,t\},x] \simeq \Omega_{10} \wedge \{x,v\} \notin E(G)\\ &\quad\wedge \{t,v\} \in E(G)) \end{aligned} $$

$$\begin{aligned} P_{12} (x,u,v,t) &\equiv (G[\!\{x,u,v,t\},x] \simeq \Omega_{12} \wedge \{x,v\} \notin E(G)\\ &\quad\wedge u< t) \end{aligned} $$

The left-hand side of these equations contains terms relating to different graphlet degrees (order *k*) of a given node *x*; the right-hand side consists of the number of common neighbours of specific nodes in all instances of some (*k*−1)-orbit representatives. The predicates (*P*_*i*_) exist to make sure every instance of *Ω*_*i*_ is accounted for exactly once. How these equations are generated is detailed in [10].

At first, Jesse saved the first equation that was generated for each orbit; the equations were generated in ascending order of the orbit number in the RHS sum. Orbit representatives of any order are numbered in such a way that adding an edge to an orbit representative will always increase its number, which will automatically result in an upper triangular system of equations [10].

As this selection has a clear bias towards lower densities in the orbit in the sum, this selection should first be made more fair. Random selection of which equation is kept for each orbit should solve this problem. And indeed: randomly selected equations are about twice as efficient as the first-come-first-served equations. This proves both that equation selection has a significant effect on Jesse’s running time and that the first sets that were used are not the most efficient. As it is unlikely that random equations are the most efficient either, which system of equations is most efficient must be determined.

#### Equations’ properties

There are many possible linearly independent sets of equations (16 possible sets for 4-graphlets, 1.5∗10^15^ sets for 5-graphlets and 3.5∗10^182^ for 6-graphlets), which makes finding an optimal set of equations hard. Therefore, some global criteria for equation selection must be used to try and find a heuristic that makes the algorithm faster, and to possibly identify the reason why some equations are more efficient than others.

Equations 1-3 illustrate the different properties equations can have. The number of terms in the left-hand side ranges from 2 in Eq. (1) to 4 in Eq. (2) to 8 in Eq. (3). The orbit in the sum in the right-hand side is orbit 6 from graphlet 4 in Eq. (1), orbit 10 from graphlet 6 in Eq. (2) and orbit 12 from graphlet 7 in Eq. (3). The number of different *c*(...) terms within the sum in the right-hand side is 1 in Eq. (2) and 2 in Eqs. (1) and (3). Note that the total number of terms is much higher, but depends on the prevalence of the orbit in the RHS sum within the explored graph. For the purpose of equation selection, only properties that can be calculated without prior knowledge of the graph will be considered. Finally, the number of arguments of each of these *c*(...) terms (i.e. the number of nodes whose common neighbours are counted) is 3 in Eq. (1), 2 in Eq. (2) and 1 in Eq. (3). As a shorthand, LHS and RHS will be used to denote the left-hand side and right-hand side of an equation, respectively, for the remainder of this paper.

The left-hand side can be safely ignored because each orbit will only be linked to denser orbits. Therefore, the system of equations will automatically be an upper triangular matrix, without any need for decomposition. The time needed to solve any single equation will therefore be *O*(*n*_Orb,*k*_), in which *n*_Orb,*k*_ is the number of different orbits of *k*-graphlets. The time to compose this equation will be *O*(*o*_*i*_(*x*)) with *o*_*i*_ an graphlet degree of some (*k*−1)-graphlet, assuming all instances of *Ω*_*i*_ have been found. As *o*_*i*_(*x*)≫*n*_Orb,*k*_ in most graphs for at least one *o*_*i*_, most effort will be needed to calculate the right-hand side of the equations. That leaves three properties: the *orbit* over whose instances the sum is made; the *number of terms* within the sum, by which the number of explicitly written *c*(...) terms is meant – not the total number of terms if the sum is expanded, which depends on the explored graph; and the *number of arguments of the terms* in the right-hand side, which means the number of nodes that are enumerated within the parentheses of each *c*(...) term.

Two of these properties, however, are related. Remember that an equation needs to be selected for each orbit, so the lowest numbered orbit in the left-hand-side of the equation is fixed. If this is fixed, though, the orbit in the RHS sum completely determines the number of arguments of the terms in the right-hand side: the number of arguments of the terms will exactly be the number of edges that must be added to the orbit in the RHS sum to create the lowest numbered orbit in the LHS. In the previous example, orbit 40 has 3 more edges than orbit 6, so the number of arguments of the terms in the RHS of Eq. (1) must be 3. Orbit 40 has only 1 edge more than orbit 12, so the terms in the RHS of Eq. (3) must have 1 argument. As such, if the size of the orbit representative in the RHS sum increases, the number of arguments of the RHS terms will stay the same or decrease. Maximising the size of the orbit representative in the RHS sum will therefore minimise the number of arguments of the RHS terms, and vice versa.

On the other hand, the number of terms in the right-hand-side of the equation is dependent on the symmetry of the orbit representative in the sum, and of the specific nodes in each term. While these are of course related to the orbit representative, that relation is not straightforwardly characterised. Therefore, the effect of this factor will need to be calculated separately.

### Random graphs used for testing

Three types of random graphs were used for this research: Erdős-Rényi graphs, Barabási-Albert graphs and geometric graphs. A short explanation of how these graphs are generated follows here.

#### Erdős-Rényi graphs

Erdős-Rényi graphs [13], or ER graphs for short, are true random graphs. Given a certain number of nodes and edges, a random graph is generated that has exactly this order and degree. Every possible edge has the same probability to exist, making the chance that any pair of nodes is connected a constant.

These graphs are generated by first creating a graph with the desired number of nodes but no edges, then randomly adding edges one by one, uniformly selecting a random unconnected pair of nodes for each edge.

#### Barabási-Albert graphs

In Barabási-Albert graphs [14], shortened to BA graphs, edges are preferentially connected to high-degree nodes. The values needed to generate a Barabási-Albert graph are its order and starting degree of each node *δ* (equal to half the average degree of the graph). Barabási-Albert graphs are scale-free graphs, meaning that the degree distribution of a Barabási-Albert graph follows a power law.

These graphs are generated starting from a connected graph with order equal to the starting degree. Any connected graph can be used as starting point; here, a complete graph was chosen to be able to get high-density BA graphs. Then, *δ* different nodes are randomly drawn from the present nodes, in which each node’s chance to be drawn is proportional to its degree. A new node is added, connected to these nodes. This is repeated until the graph has the required order.

#### Geometric graphs

Geometric graphs [15] are characterised by the fact that their nodes have a spatial position. This position might be in any number of dimensions (*D*). Pairs of nodes that are within a certain threshold distance of each other are connected. This threshold distance is a parameter that is determined at the start of the graph generation.

To generate these graphs, all needed nodes are positioned randomly within a *D*-dimensional cube of unit size. When each node is connected, it is connected to the present nodes within the threshold distance from that node. Note: for some experiments, in particular those in which the speed of Jesse is plotted as a function of a graph’s density, the nodes were distributed in a *D*-dimensional torus instead, so coordinate 1 on any axis is equal to coordinate 0. Most of the time, *D*=3 in the following experiments.

## Results

### Effect of equation selection

The effects of equation selection were striking. In a first test, graphlets of 4, 5, 6 and 7 nodes were counted in Erdős-Rényi graphs with 50 nodes and 500 edges. Five selections of equations were used: random equations, equations with the fewest possible right-hand side terms, equations with the most possible right-hand side terms, equations with right-hand side terms with the fewest possible arguments and equations with right-hand side terms with the most possible arguments. The result of these measurements can be seen in Table 1.

**Table 1:** The running time of Jesse with different equations in ER graphs of order 50 and size 500, for graphlets of different orders

	Random equations	Fewer RHS terms	More RHS terms	RHS terms with fewer arguments	RHS terms with more arguments
4	0.018 ± 0.004	0.017 ± 0.004	0.017 ± 0.004	0.017 ± 0.004	0.015 ± 0.003
5	0.32 ± 0.02	0.32 ± 0.01	0.30 ± 0.02	0.22 ± 0.01	0.36 ± 0.01
6	11.0 ± 0.3	10.9 ± 0.2	10.5 ± 0.3	6.9 ± 0.2	15.6 ± 1.4
7	283 ± 13	278 ± 20	279 ± 20	180 ± 3	380 ± 7

All measurements were repeated 20 times. The average and standard deviation of the resulting running times is shown

The number of arguments has a large influence on Jesse’s running time – up to almost a factor 2 – but selecting equations based on the number of terms does not seem to influence Jesse’s running time. Therefore, in all further experiments, equations will be selected based on the number of arguments in each RHS term.

#### Cause

All possible *c*(...) terms, i.e. the number of common neighbours of all sets of up to *k*−2 nodes, are calculated before the graphlets are searched. Then, these are stored in a hash map with the arguments of each term as the key. When calculating the equations’ right-hand sides, the *c*(...) terms are looked up from there. As the equations’ right-hand side consists completely of a sum of these terms, this is the most logical place to start looking for the determining factor that influences the speed.

In Jesse, unlike in ORCA, all possible *c*(...) terms are stored in a single hash map. In ORCA, these are saved in multiple hash maps, a different hash map for each set size (i.e. the number of arguments of the RHS terms). So all common neighbours of 2 nodes are saved in a map, the common neighbours of 3 nodes in another, and so forth. To test whether this approach made a significant difference in running time, Jesse was temporarily adapted to save the *c*(...) terms in different maps. However, saving them in different maps did not make the algorithm run significantly faster than saving them in the same map. This indicates that the time difference between looking up small sets of nodes and large sets is not related to hash tables that possibly have too many collisions to be used well.

Secondly, Jesse was adapted to only use the hash codes of the sets of nodes as keys in the hash map. Note that in Java, hash maps can distinguish between different key objects with the same hash code. To do so, the entire key objects must be compared. Explicitly using the hash codes of the arguments of the *c*(...) terms as keys disabled the comparison of those lists. While this did speed up the algorithm with all sets of equations – in exchange for wrong results in large graphs because of hash collisions – it did not remove the speed difference. This indicates that the longer runtime of Jesse when looking up terms with more arguments is not due to the repeated comparison of longer lists to each other, although comparing lists has an effect on Jesse’s overall speed.

**Fig. 2:**
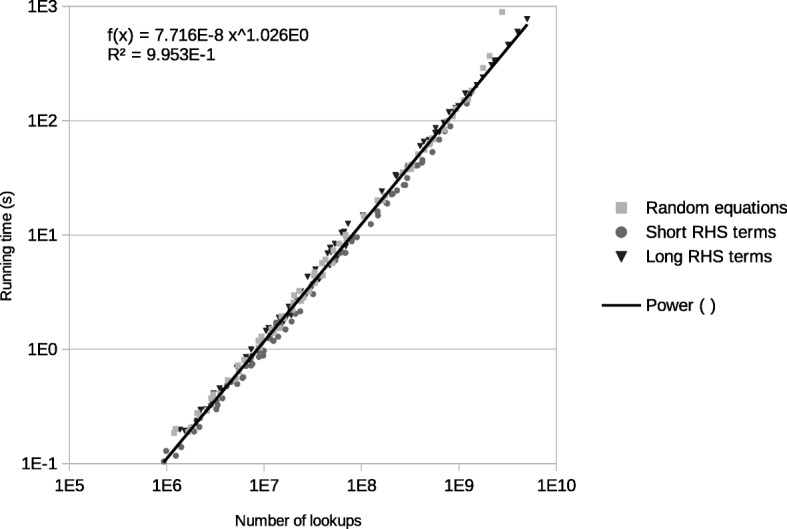
All datapoints from all runs of the speed vs number of lookups test. The datapoints are in grayscale, according to the set of equations that was used to generate them. On the x axis: the number of lookups done, on the y axis: the running time in seconds. The datapoints were fitted with a power trend line of *f*(*x*)=7.72∗10^−8^*x*^1.03^

The two previous tests suggest that the time per lookup in the hash table is not too different between small and large sets of arguments. Therefore, the logical next step was to log the number of lookups that were used in runs with different equation sets. And indeed, there was a large difference in the number of lookups done between the best and worst sets of equations. Jesse was run with different sets of equations (random, right-hand side terms with fewest arguments and with most arguments) for different graphlet orders (5 and 6) on different graph types (Erdős-Rényi, Barabási-Albert and geometric) of different orders (50 to 200 nodes) and sizes (average degree 16 to 24). In all of these cases we recorded the time needed to calculate the graphlet degrees, as well as the number of lookups done. All of the generated data together can be seen in Fig. 2.

Even though all of these data points come from different graph types, with different orders and sizes; different graphlet orders; and equations that were selected in different ways, a power law fits them surprisingly well. Therefore, it seems safe to assume that the lookups are the largest cause of the speed difference between different sets of equations. However, the fit is a power law with an exponent that is close to, but different from 1. This may be due to other effects than lookups becoming more important at higher or lower density. The data points are coloured according to the used equations (Fig. 2), so it can also be seen that the datapoints made with the most efficient equations tend to be under the fitted line, whereas the datapoints using the least efficient equations are above the fitted line. This indicates that, apart from the number of lookups, there must be other factors making these equations more efficient. However, the number of lookups is clearly the most important factor for Jesse’s speed and looks like the most promising factor to optimize.

#### Reason for higher equation efficiency

In previous sections, it was illustrated that the equation set that results in the smallest number of lookups of common neighbours will be the most efficient set. However, we did not yet offer an explanation of why the equations with RHS terms with the fewest number of terms tend to be the ones with the fewest lookups in about all used graphs.

Recall that, for a fixed lowest numbered left-hand side orbit, the number of arguments of the RHS terms is directly related to the number of edges in the orbit representative over which the sum in the RHS is made. Terms with fewer arguments mean denser graphlets, which appear less frequently in a relatively sparse graph. In an ER graph, the expected number of instances of each orbit representative can even be calculated analytically fairly easily. In an ER graph *G* with *n* nodes and *m* edges, an orbit representative *Ω* with *k* nodes and *e* edges will appear

$$\prod\limits_{i=0}^{e-1} \frac{m-i}{\binom{n}{2}-i} * \prod\limits_{i=e}^{{k \choose 2}-1} \frac{\binom{n}{2}-m-i}{\binom{n}{2}-i} * \binom{n}{k} * S$$

times, with $S = \frac {k!}{|\text {Aut}(\Omega)|}$. If *k*≪*m* and *k*≪*n*, this reduces to

$$(D(G))^{e} * (1-D(G))^{{k \choose 2} -e} * \binom{n}{k} * S, $$

in which *D*(*G*) is *G*’s density.

Leaving all other variables constant, the number of instances of a specific orbit representative increases as a function of *e* at density *D*(*G*)<0.5, stays constant at *D*(*G*)=0.5 and decreases with increasing *e* at *D*(*G*)>0.5. If the number of lookups determines the speed of the algorithm, the same behaviour is expected from the algorithm’s speed.

**Fig. 3:**
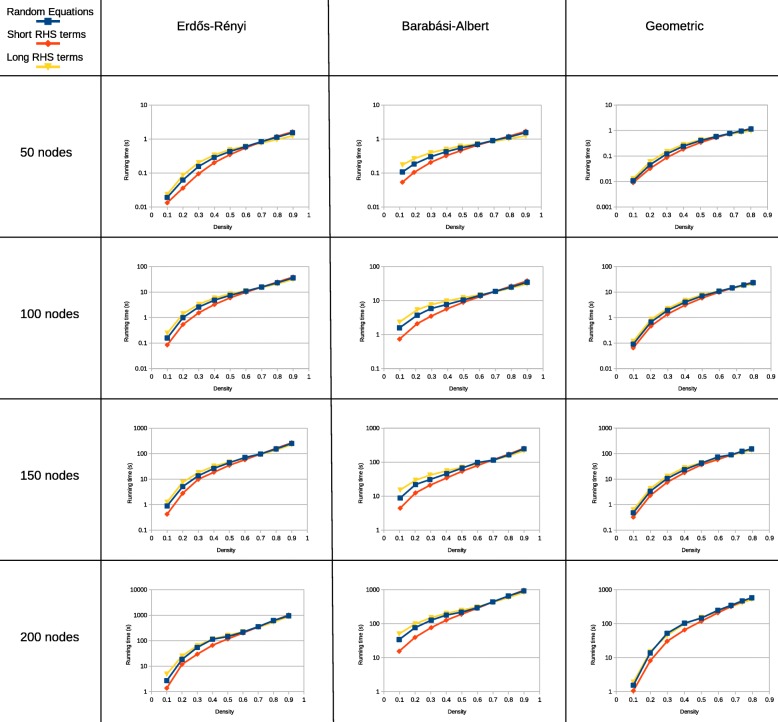
The running time of Jesse, counting 5-graphlets in ER, BA and GEO graphs of different orders and densities. Three different sets of equations were tested: randomly chosen equations, equations with RHS terms with fewer arguments and equations with RHS terms with more arguments. All experiments were repeated 20 times. The error bars show the standard deviation of all runs

**Fig. 4:**
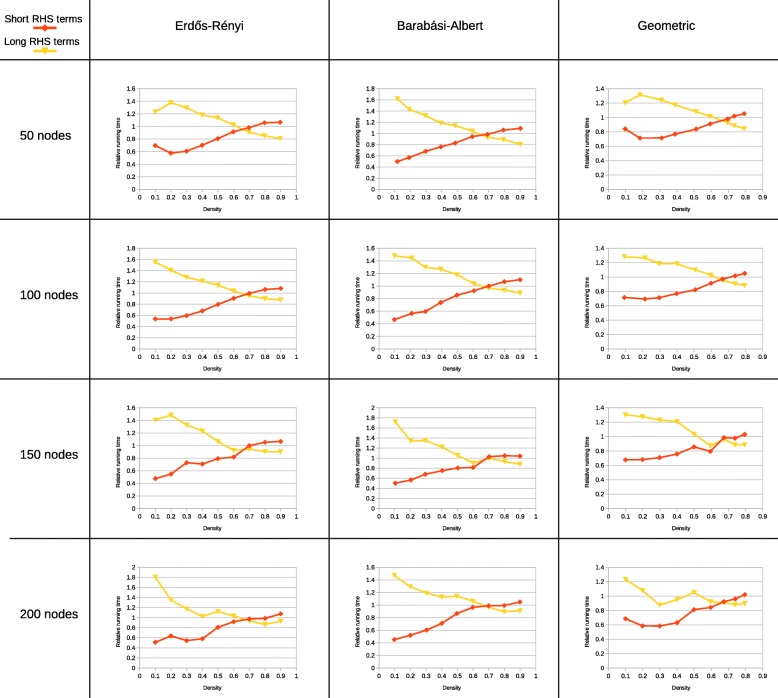
The relative running time of Jesse, counting 5-graphlets in ER, BA and GEO graphs of different orders and densities. The speed of equations with RHS terms with more or fewer arguments relative to random equations is shown, as compared to the running time with random equations. All experiments were repeated 20 times. The error bars show the standard deviation of all runs

To test this assumption, the running speed of Jesse counting 5-graphlets was measured in ER, BA and GEO graphs with densities going from 0.1 to 0.9. This was repeated for graphs of order 50, 100, 150 and 200. Each experiment was repeated 20 times. The choice for 5-graphlets was made because 5-graphlets have enough effect of equation selection, but still can be counted quickly enough to do these repetitions in reasonable time. Jesse’s speed was measured with 3 sets of equations: random equations, equations with RHS terms with fewer arguments and equations with RHS terms with more arguments. The result can be seen in Figs. 3 and 4.

While the exact shape of the curves is slightly different between the different graph types that were used, some common trends can be discerned. It can clearly be seen that the running time of the equations with RHS terms with fewer arguments increases with increasing density, whereas the running time of the equations with RHS terms with more arguments decreases with increasing density.

At a density around 0.7, the equations with RHS terms with more arguments become more efficient than the equations with RHS terms with fewer arguments. This is especially notable because this point is dependent on neither the graph type, nor the graph order. Across all tested graphs, the crossover point remains constant.

At minimal density, the running time reached by equations with RHS terms with fewer arguments is reduced by a factor 2 compared to randomly selected equations. At low density and low order, the difference in running time decreases again, because the total number of graphlets decreases. Therefore, the relative amount of time used by the tree-walking graphlet search, which is independent of the equations, becomes larger with respect to the time spent looking up common neighbours. At maximal density, the running time of equations with RHS terms with more arguments is about 10% shorter than the running time with randomly selected equations. Because of the longer running time at high density, the absolute difference in running time is larger for denser graphlets.

For almost all graphs, the running time with random equations is close to the average of the running time of equations with RHS terms with more arguments and RHS terms with fewer arguments. At medium density, they are sometimes less efficient than either (for instance in geometric graphs around a density of 0.6). That seems to indicate that in these graphs, medium density graphlets are less common than both low and high density graphlets.

### Application to biological networks

Jesse was also benchmarked on two biological interaction networks from the IntAct database [16]: the diabetes network (169 nodes, 429 edges) and the affinomics network (1117 nodes, 2432 edges). The orbits of 6-graphlets were counted. Three versions of equation selection were used: the first-come-first-served equation selection that Jesse used before actual equation selection was implemented, random equations and equations with RHS terms with the fewest possible arguments. The choice for RHS terms with few arguments was made because these networks have a low density (0.031 and 0.0065, respectively). The speed of these runs can be seen in Table 2.

**Table 2:** The running time of Jesse counting orbits of 6-graphlets with different equations in biological interaction networks

Network	Old equations	Random equations	Few arguments
Diabetes	5.25 s	3.86 s	2.45 s
Affinomics	2.90 s	2.77 s	2.34 s

The old equations are those that were used with Jesse before equation selection was implemented

As can be seen, the original equations used by Jesse perform worst in both cases. In the diabetes network, changing to equations with short RHS terms reduces the running time by a factor of 2. The difference in the affinomics network is much less pronounced, but still present. At the extremely low density of the affinomics network, graphlets on 6 nodes become rare, which reduces the difference equations can make. The same effect can be seen in the random graphs on 50 nodes in Fig. 4.

## Discussion

Jesse is openly available at https://github.com/biointec/jesse as a Java command line tool. Equation selection is now implemented: Jesse will automatically select the most appropriate set of equations. When analysing graphs with density below 0.7, equations with RHS terms with fewer arguments will be used; for graphs with a density above 0.7, equations with RHS terms with more arguments will be used. It is possible to select other equations, however. This might be useful in disconnected graphs in which every connected component has a density above 0.7, but the entire graph has a density below 0.7, for instance.

### The Jesse Cytoscape app

**Fig. 5:**
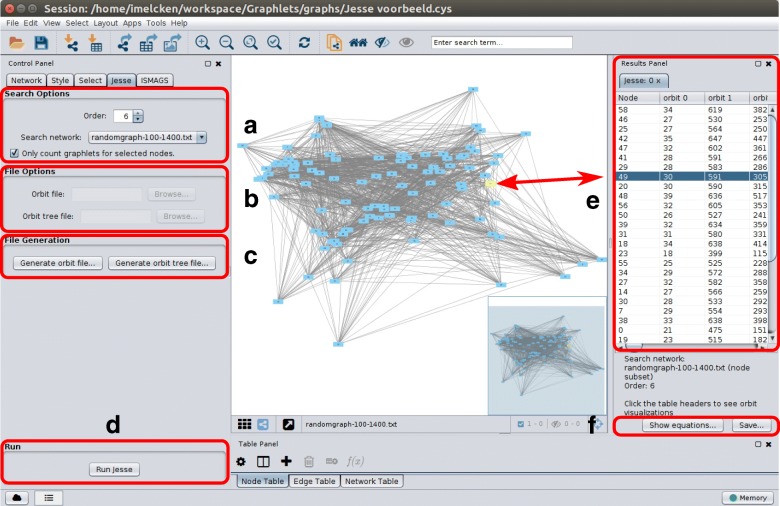
The Jesse interface in Cytoscape. **a** Search Options panel, with basic options (graphlet order, search network, only count graphlets for selected nodes). **b** File Options panel, used to select orbit and tree files for graphlets of order greater than 7. **c** File Generation panel, used to generate orbit and tree files for graphlets of order greater than 7. **d** Run button to start the algorithm. **e** Results panel, where the graphlet degrees of each node can be seen. Selecting a node in the network view will also select it in the results panel, and vice versa, as indicated by the arrow. Clicking on a column header will visualise the corresponding orbit. **f** Buttons to save the used equations and computed graphlet degrees

For ease of use, all of Jesse was integrated in a Cytoscape app. This app, named Jesse, can be found on the Cytoscape App store here: http://apps.cytoscape.org/apps/jesse. The interface of this app can be seen in Fig. 5.

It adds a new tab to Cytoscape’s control panel, named Jesse. In this tab, the box titled *Search Options*, marked with the character A in Fig. 5, contains the most basic options for orbit counting. The largest order of graphlets whose orbits need to be counted can be selected here, as well as any opened Cytoscape network, in which the orbits will be counted. A checkbox can be ticked to compute the graphlet degrees of only the nodes that are selected in the network view, otherwise the graphlet degrees of all nodes in the network are calculated. If the selected order is between 3 and 7 (included), then no other options are needed. Just pushing the *Run* button, character D in Fig. 5, at the bottom of the control panel is enough.

If the order of graphlets that need to be counted is greater than 7, no standard files for orbit counting are included with Jesse. They can, however, be generated for any order using the *File Generation* box, character B in Fig. 5. Files for both orbit numbering and the used search tree need to be generated and saved. Once they are generated, the files for a given graphlet order can be used anytime the Jesse app is used, without generating them again. Choosing generated files can be done in the *File Options* box, character C in Fig. 5.

When the algorithm has finished running, the *Results* panel, character E in Fig. 5, appears on the right-hand side of the Cytoscape window. There, a table showing each node’s graphlet degrees can be seen. Clicking on a node’s name will highlight it in the network view; conversely, highlighting a node in the network will show its graphlet degrees. This allows quick inspection of a specific node’s graphlet degrees. Clicking on an orbit’s number in the table header will open a new network showing a visualisation of that orbit representative in a direct and intuitive way.

The used system of equations, as well as the results table, can be exported using the buttons at the bottom of the Results panel, character F in Fig. 5. The equations can be saved in pdf format or as plain text, TeX code or png, the results in a text file. Each line of the text file starts with the name of a node, followed by the graphlet degrees ordered by orbit number, separated by tabs. This allows post-processing of these data with any other tools the user may have at their disposal.

### Future work

The sets of equations that were tested in this paper were selected based on global criteria. They were meant to minimise the number of lookups that have to be done without prior knowlegde of the explored graph’s structure. Especially in graphs with a more complicated small-scale structure than the ones that were tested here, it is possible that the prevalence of a certain orbit representative does not depend solely on its density. Therefore, even in a sparse graph, it may be possible that a certain sparse graphlet appears less often than a dense graphlet. Then, selecting equations based on the RHS term number of arguments will not result in the best equation set.

If the number of orbit representatives of order *k*−1 is counted before applying the equations, as opposed to applying the equations during counting, the number of lookups may be calculated for each equation. For every orbit, the equation that will need the fewest lookups can then be chosen. However, then the instances should be saved, because the instances need to be filled in the equations, which in itself causes an overhead.

As the orbits are counted for every node separately, it may be entirely possible that the best equations are different from node to node in a single graph. Therefore, an additional speedup might be achieved by tailoring the equations to every node. It remains to be seen whether this difference is significant and outweighs the effort needed to calculate which equations to use.

## Conclusion

With the addition of equation selection, the bioinformatics network analysis tool Jesse has been significantly sped up. Which equations are most efficient to use is mostly dependent on the graph’s density and less on the exact type of graph that is used. At low graph density, equations with right-hand side terms with fewer arguments are most efficient, being up to twice as fast as random equations and four times faster than equations with right-hand side terms with more arguments. At a density above 0.7, equations with right-hand side terms with more arguments become more efficient, reaching a running time reduction of about 10% compared to random equations at extremely high density. The number of terms in equations’ right hand side has no significant effect on the algorithm’s running time.

The difference in running time when using different sets of equations can be attributed to the vastly different total number of terms that make up the equations’ right hand sides. These terms need to be looked up in a hash table, and the number of lookups needed has the dominant effect on the different running time between equations.

This knowledge was implemented into Jesse: it now automatically selects a system of equations based on the given graph’s density. When its density is lower than 0.7, the equations with the right-hand side terms with the fewest arguments are chosen, when its density is higher than 0.7 those with the right-hand side terms with the most arguments are chosen. A Cytoscape App for Jesse was created, which combines Jesse’s functionality with Cytoscape’s user-friendly interface and can be downloaded from the Cytoscape App Store.

## Availability and requirements

Jesse as a command line tool is freely available at Github (https://github.com/biointec/jesse). It is implemented in Java, therefore it is platform-independent. It needs JRE 1.8.

As a Cytoscape App, Jesse can be downloaded from the Cytoscape App Store (http://apps.cytoscape.org/apps/jesse) and needs Cytoscape 3.x.

## Abbreviations

BA: Barabási-Albert (random graph)
ER: Erdős-Rényi (random graph)
GDD: Graphlet degree distribution
GEO: Geometric (random graph)
LHS: Left-hand Side (of an equation)
RHS: Right-hand Side (of an equation)
